# Single‐cell transcriptomics reveal the remodeling landscape of bladder in patients with obstruction‐induced detrusor underactivity

**DOI:** 10.1002/mco2.490

**Published:** 2024-02-26

**Authors:** Jiawei Chen, Liao Peng, Guo Chen, Yuanzhuo Chen, Xiao Zeng, Jie Zhang, Chi Zhang, Hong Shen, Banghua Liao, Deyi Luo

**Affiliations:** ^1^ Department of Urology West China Hospital Sichuan University Sichuan China; ^2^ Department of Urology, Institute of Urology West China Hospital Sichuan University Sichuan China; ^3^ Department of Urology and Pelvic surgery West China School of Public Health and West China Fourth Hospital Sichuan University Sichuan China; ^4^ Pelvic Floor Diseases Center West China Tianfu Hospital Sichuan University Sichuan China

**Keywords:** bladder outlet obstruction, bladder remodeling, detrusor underactivity, single‐cell RNA sequencing

## Abstract

Detrusor underactivity (DUA) is a common and thorny problem in urology, which severely impairs patients’ bladder function and quality of life. However, its underlying pathophysiological mechanism remains unclear. Hence, we sequenced 69,973 cells from five controls and nine patients with bladder dysfunction using single‐cell RNA sequencing. Twelve distinct cell types were identified and they showed high cellular and functional heterogeneity among each group. Among them, fibroblasts, macrophages, and epithelial cells had the most intercellular communications. Their aberrant gene expressions and altered intercellular interactions were mainly involved in extracellular matrix organization, inflammation/immune regulation, and cellular injury. Further re‐cluster analysis revealed an accumulation of the RBFOX1^+^ fibroblasts and RIPOR2^+^ macrophages in dysfunctional bladder wall, which mediated bladder remodeling through dysfunctional extracellular matrix organization and inflammation/immune reaction. Besides, the subtype of the epithelial cells was significantly altered. They underwent an intricate process including inflammation, damage, and repair during bladder remodeling. Overall, this work constructed the first single‐cell atlas for obstruction‐induced DUA, which could provide a valuable resource for deciphering the cellular heterogeneity and function changes in DUA, as well as potential strategies for bladder function improvement.

## Graphical abstract

This study constructed the first single‐cell landscapes for obstruction‐induced detrusor underactivity and identified 12 distinct cell types in the remodeling bladder. Furthermore, we revealed that cellular heterogeneity and the alteration of gene expression and cell–cell interactions in obstruction‐induced detrusor underactivity. Re‐cluster analysis revealed that the *RBFOX1* ^+^ fibroblast, *RIPOR2* ^+^ macrophage, and inflammatory epithelial cell could be the key cells mediating obstruction‐induced bladder remodeling through extracellular matrix organization and inflammation regulation.

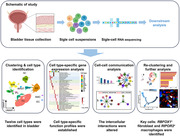

## 1 INTRODUCTION

Detrusor underactivity (DUA) is a common but thorny urologic problem for patients in which contraction strength and/or duration of bladder detrusor reduced, resulting in prolonged bladder emptying and/or failure to achieve complete bladder emptying within a normal time span,^1^ causing bothersome lower urinary tract symptom (LUTS). DUA significantly decreased patients’ quality of life (QoL)^2^ and led to adverse consequent effects on health such as recurrent urinary tract infections, urinary retention, and even renal impairment. Due to lacking an easily and accurate measurable proxy, the epidemiology of DUA is poorly understood.^3^ Still, the estimated prevalence is about ranging from 9% to 48% among different population, and such prevalence would increase with age.^3^, ^4^ Despite its high prevalence and severe disease burden, DUA receives limited research invested in promoting a comprehensive understanding of its pathophysiological mechanism.

The etiology of DUA is multifactorial, primarily classified into two main categories: abnormality of detrusor itself (myogenic) and/or neural control (neurogenic).^3^, ^5^ Among them, bladder outlet obstruction (BOO) is a ubiquitous cause for DUA. Patients with BOO need an increased detrusor pressure to offset increased resistance of bladder outlet and void completely, and the resulting long‐term intravesical pressure overload leads to a change in the structure and function of the bladder, known as bladder remodeling.^6^ It is a chronic process from compensation to decompensation, characterized by initial inflammation, subsequent smooth muscle hypertrophy, and extracellular matrix (ECM) excessive accumulation to eventual fibrosis.^6^ Functionally, as such progress of remodeling, the corresponding urodynamic pattern will change from “high pressure with low flow” to “low pressure with low flow,” reflecting the reduced detrusor contractility.^7^ As DUA is the irreversible consequences of myogenic and neurogenic impairments of prolonged BOO, many storage and voiding symptoms remain after surgical relief of obstruction. In the clinical practice, for such patients, there is no consensus on whether they are appropriate candidates for transurethral surgery because DUA has been reported to correlate with poorer symptom improvement after surgery.^8^ Hence, there is an urgent need to elaborate the pathophysiological courses and molecular mechanism of BOO‐induced DUA and find potential targets to avoid even reverse bladder decompensation even failure.

Previous studies have done much work on bladder smooth muscle cells in bladder remodeling from the aspects of morphological, functional, and molecular changes.^9^, ^10^ In fact, the cellular composition of the bladder is complex. Besides smooth muscle cells, it additionally comprises non‐immune cell (like urothelium, endothelium, and fibroblasts) and immune cells (like T cells, B cells, and macrophages).^11^ Although more and more studies have begun to pay attention to the roles of other cell types in bladder remodeling, such as urothelium^12^ and macrophage,^13^ they only focused on a certain type of bladder cell but ignored cellular heterogeneity and intercellular interactions. Moreover, to understand the molecular mechanism of the remodeling process, it is also important to investigate the changes of cellular composition and gene expression in normal and remodeled bladder walls.

Single‐cell RNA sequencing (scRNA‐seq) is a powerful technology, which enables to dissect profiling of cellular composition and gene expression at single cell resolution.^14^ Yu et al. applied scRNA‐seq to create a single‐cell transcriptomic map of human and mouse normal bladders.^11^ Hence, using scRNA‐seq, our study aims to provide a comprehensive transcriptomic atlas of BOO‐induced remodeled bladder at a single cell level, which could offer an in‐depth insight for pathophysiological process of BOO‐induced bladder dysfunction and promising targets for preventing or treatment of this disorder.

## 2 RESULTS

### 2.1 Single‐cell transcriptomic atlas of obstruction‐induced bladder dysfunction

**FIGURE 1:**
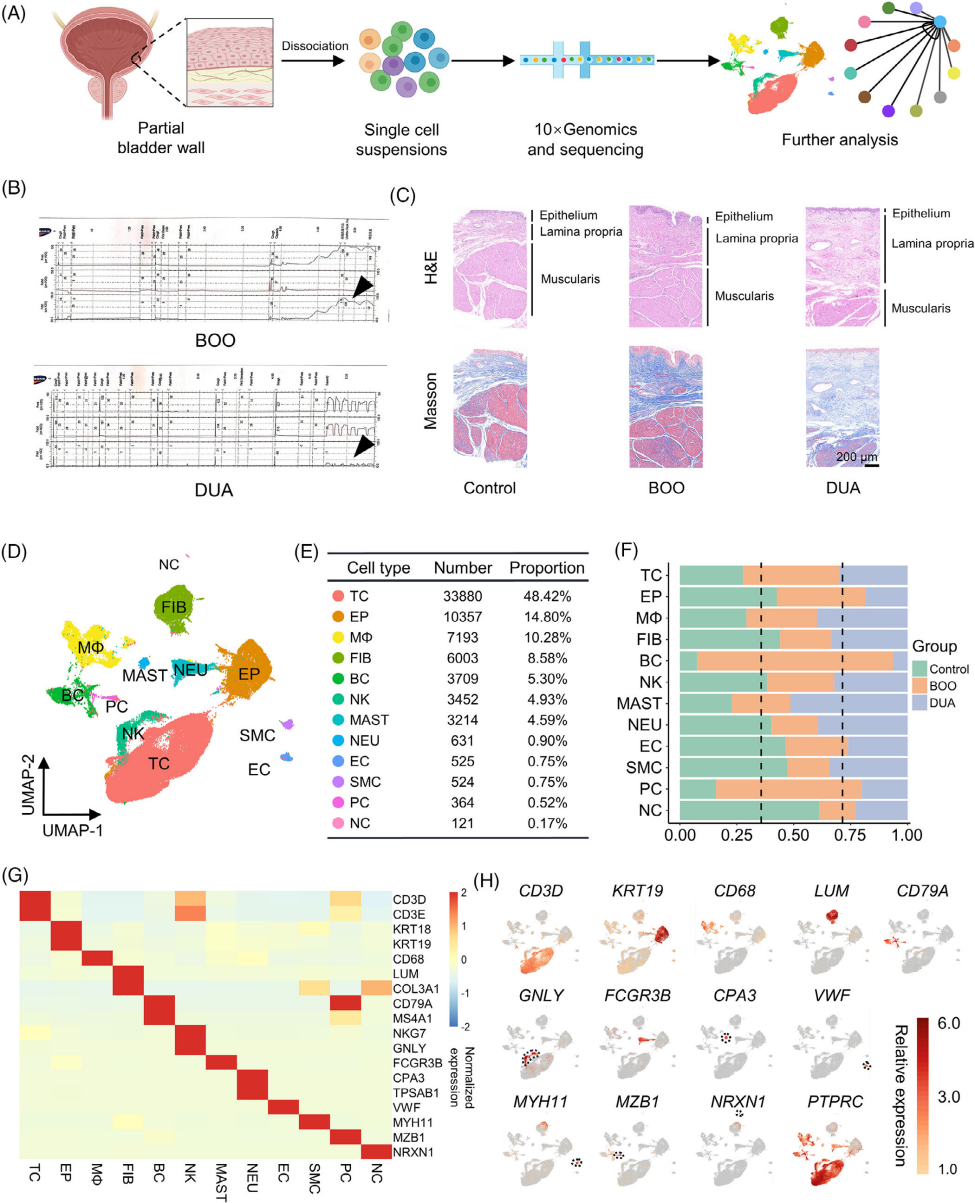
Single‐cell atlas of normal and dysfunctional bladder wall. (A) Schematic of tissue dissociation, cell isolation, single‐cell RNA sequencing, and further bioinformatics analysis. (B) Representative urodynamic results of bladder outlet obstruction (BOO) and detrusor underactivity (DUA). Black arrows point the pressure of detrusor. (C) Representative H&E and Masson staining of bladder from control, BOO, and DUA groups. (D) Uniform Manifold Approximation and Projection (UMAP) plots of the 12 identified cell types of bladder tissue. TC, T cell; EP, epithelial cell; MΦ, macrophage; FIB, fibroblast; BC, B cell; NK, nature kill cell; NEU, neutrophil; MAST, mast cell; EC, endothelial cell; SMC, smooth muscle cell; PC, plasma cell; NC, neural cell. (E) The number and proportion of each cell types. (F) Bar plot shows the proportion of each cell types in the three groups, colored according to different group, green, control group; orange, BOO group, and purple, DUA group. Dashed black line represents the expected proportion of cells in each group (total number of cells in each group divided by total number of cells from all specimens). (G) Heatmap shows relative expression of several marker genes in each cell types, which are color coded based on their normalized expression on the right. (H) UMAP plot displays relative expression of several marker genes in each cell types, which are color‐coded based on their relative expression on the right.

To explore the underlying mechanism of obstruction‐induced bladder dysfunction, scRNA‐seq and subsequent bioinformatics analysis was performed using bladder single cells isolated from the five control, five BOO, and four DUA individuals according to the standard methods of 10× Chromium Genomics protocols (Figure 1A). Patients in the BOO group exhibited a characteristic high‐pressure, low‐flow voiding pattern, whereas those in the DUA group demonstrated a typical low‐pressure, low‐flow pattern of urination (Figure 1B). Their baselines characteristics, scores on symptom‐related scales, and urodynamic parameters are summarized in Table 1 and Table S1. The histological and morphological features were visualized using hematoxylin and eosin (H&E) staining, while collagen deposition was demonstrated through Masson's trichrome staining (Figure 1C). As expected, the dysfunctional bladder had notable alterations in histological morphology compared with the control group. The bladder from patients with BOO and DUA displayed a significant thickening of the lamina propria, particularly in the case of the DUA group. Additionally, the lamina propria and muscle layer demonstrated an abundant deposition of collagen. Such morphological changes were consistent with the findings previously reported.^4^, ^10^

**TABLE 1:** Baseline characteristics and preoperative International Prostate Symptom Score (IPSS) and quality of life (QoL) of participants.

Variable	Control (*n* = 5)	BOO (*n* = 5)	DUA (*n* = 4)	*p* values
Age, years	55.4 ± 3.1	66.6 ± 7.4	70.0 ± 3.9	0.024^a^
Body mass index, kg/m^2^	22.3 ± 0.8	22.0 ± 0.4	22.9 ± 1.2	0.396^a^
Time since LUTS, years	–	1.9 ± 0.4	4.5 ± 0.5	<0.001^b^
Smoking, *n* (%)	0 (0.0)	0 (0.0)	0 (0.0)	–
IPSS	4.0 ± 2.6	21.4 ± 1.7	22.5 ± 2.1	<0.001^a^
QoL	1.2 ± 0.8	4.4 ± 0.9	4.5 ± 0.6	<0.001^a^
BOOI	–	83.4 ± 16.4	9.8 ± 3.9	<0.001^a^
BCI	–	138.0 ± 16.5	39.5 ± 19.2	<0.001^a^

Abbreviations: BCI, bladder contractility index; BOOI, bladder outlet obstruction index; IPSS, International Prostate Symptom Score; LUTS, lower urinary tract symptoms; QoL, quality of life.

^a^ One‐way analysis of variance test.

^b^ Two‐tailed unpaired Student's *t*‐test.

After quality control, a total of 69,973 cells were deemed suitable for downstream analysis, with 21,747 (31.08%) cells from the control group, 27,548 (39.37%) cells from the BOO group, and 20,678 (29.55%) cells from the DUA group. Thirty clusters were initially yielded after dimensionality reduction using the PCA and visualized through Uniform Manifold Approximation and Projection (UMAP). According to the canonical markers among the top expressed genes of each cluster (Figure 1G,H and Figure S1A), they were merged by similar gene expression profiles and annotated as 12 major cell types, included seven types of immune cells (T cells, macrophages, B cells, natural killer cells, neutrophils, mast cells, and plasma cells) and five types of nonimmune cells (epithelial cells, fibroblasts, endothelial cells, smooth muscle cells, and neural cells) (Figure 1D,E). The two most abundant immune cell types were T cells (48.42%) and macrophages (10.28%), and nonimmune cells were epithelial cells (14.80%) and fibroblasts (8.58%) (Figure 1E). Moreover, gene ontology (GO) analysis based on the highly expressed genes in each cell types further confirmed the accuracy of the cell identification (Figure S1B). The function of each cluster was consistent with its annotation. For example, the cluster designated as “fibroblast” exhibited a significant enrichment of highly expressed genes associated with ECM functions. The top‐expressed genes of cluster named as “macrophage” were enriched in the phagocytosis and chemotaxis. As for the heterogeneity of cellular composition, T cells, epithelial cells, fibroblasts, and macrophages constituted the predominant cell types in each group. Notably, epithelial cell was gradually decreased from control to DUA, and B cell was found to be abundant in the BOO group (Figure 1F and Figure S1C). Collectively, we constructed a comprehensive single‐cell transcriptomic atlas and elucidated the cellular composition of obstruction‐induced bladder dysfunction.

### 2.2 Cell type‐specific gene expression and function profiles in the dysfunction bladder

**FIGURE 2:**
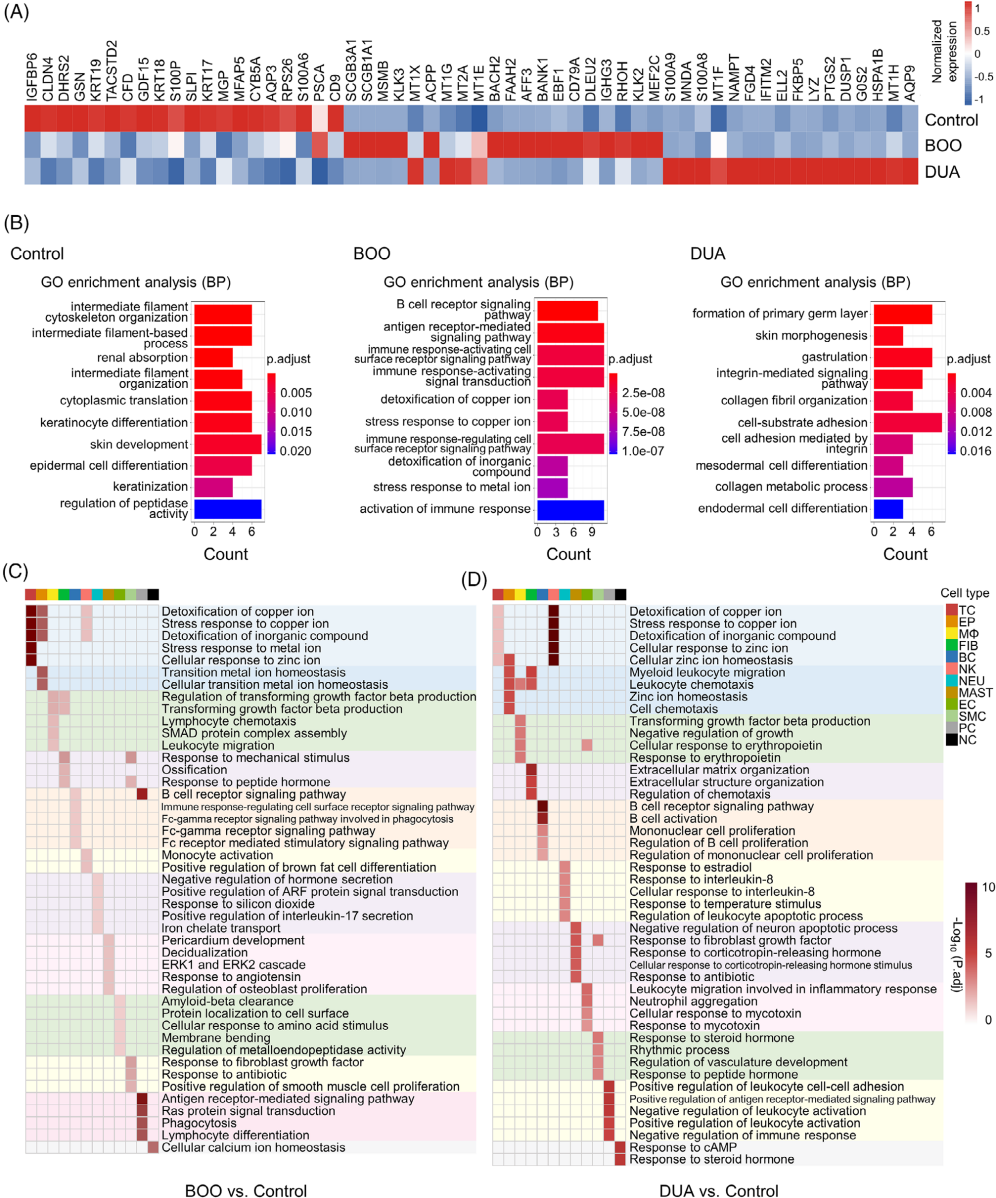
Cell‐specific gene expression and function profiles in the dysfunction bladder. (A) Heatmap shows the relative expression of top 20 differentially expressed genes among three groups. (B) Gene ontology (GO) enrichment analysis based on the differentially expressed genes, colored according the significance. BOO, bladder outlet obstruction; DUA, detrusor underactivity; BP, biological process. (C and D) Cell‐specific GO enrichment analysis (biological process) according to the upregulated differentially expressed genes of BOO (C) or DUA (D) compared with control, color‐coded by the significance of each term. Colors on the top represent cell types (right). TC, T cell; EP, epithelial cell; MΦ, macrophage; FIB, fibroblast; BC, B cell; NK, nature kill cell; NEU, neutrophil; MAST, mast cell; EC, endothelial cell; SMC, smooth muscle cell; PC, plasma cell; NC, neural cell.

To profile the changes of gene expression and function, enrichment analysis was performed at both group and cellular levels. From control to DUA, each group showed a distinct expression and function pattern of differently expressed genes (DEGs) (Figure 2A,B). In the control group, the majority of the top 20 DEGs were associated with the epithelial cells, including epithelial barrier‐related genes (CLDN4, KRT19, and KRT18), and SLPI, a key gene reported to be localized to bladder epithelial cells and protect against urinary tract infection in mouse model.^15^ Also, the GO enrichment analysis confirmed that the functions of such DEGs were related to the epithelial function (Figure 2B). As for the BOO group, interestingly, the top two DEGs, SCGB3A1 and SCGB1A1, which belong to the secretoglobin family, have been reported to play a crucial role in immune regulation and could potentially serve as biomarkers for lung tissue damage caused by mechanical ventilation.^16^ The presence of these two markers could indicate the bladder injury attribute to the overload of intravesical pressure. The GO enrichment analysis revealed that the immune‐related process was upregulated in the BOO group (Figure 2B). For DEGs of the DUA group, most of them were primarily involved in inflammation and immune regulation, such as S100A8, S100A9, and MNDA.^17^ Interestingly, the function of all DEGs in DUA was enriched in the organ fibrosis and remodeling, like integrin and collagen‐related biological processes (Figure 2B).

Furthermore, a cell‐type specific enrichment analysis based on both up‐ or downregulated DEGs were performed to evaluate the alterations of function in each cell types (Figure 2C,D and Figure S2). Compared with the control group, the upregulated DEGs of macrophages in the both BOO and DUA group were involved in transforming growth factor beta (TGF‐β)‐related pathway, a critical mechanism governing organ healing and fibrosis.^18^ Notably, fibroblasts of the BOO group exhibited a response to the mechanical stimulus—the characteristic pathophysiological changes resulting in the high intravesical pressure after BOO (Figure 2C). While, compared to the control group, those fibroblasts in the DUA group participated in immune regulation and ECM organization (Figure 2D). However, the enrichment analysis of downregulation DEGs in both BOO and DUA group appeared to yield some inconclusive findings (Figure S2).

Taken together, these results suggested that obstruction‐induced bladder dysfunction might involve a comprehensive process encompassing tissue injury, inflammatory response, immune regulation, and ECM organization and remodeling, which was consistent with the previous observation in rat models.^6^ Moreover, fibroblasts were speculated to play a pivotal role in obstruction‐induced dysfunction as they were capable of responding to mechanical stimuli and actively participating in immune regulation and ECM organization.

### 2.3 Cell–cell communication analysis revealed crucial cellular types and functions in bladder dysfunction

**FIGURE 3:**
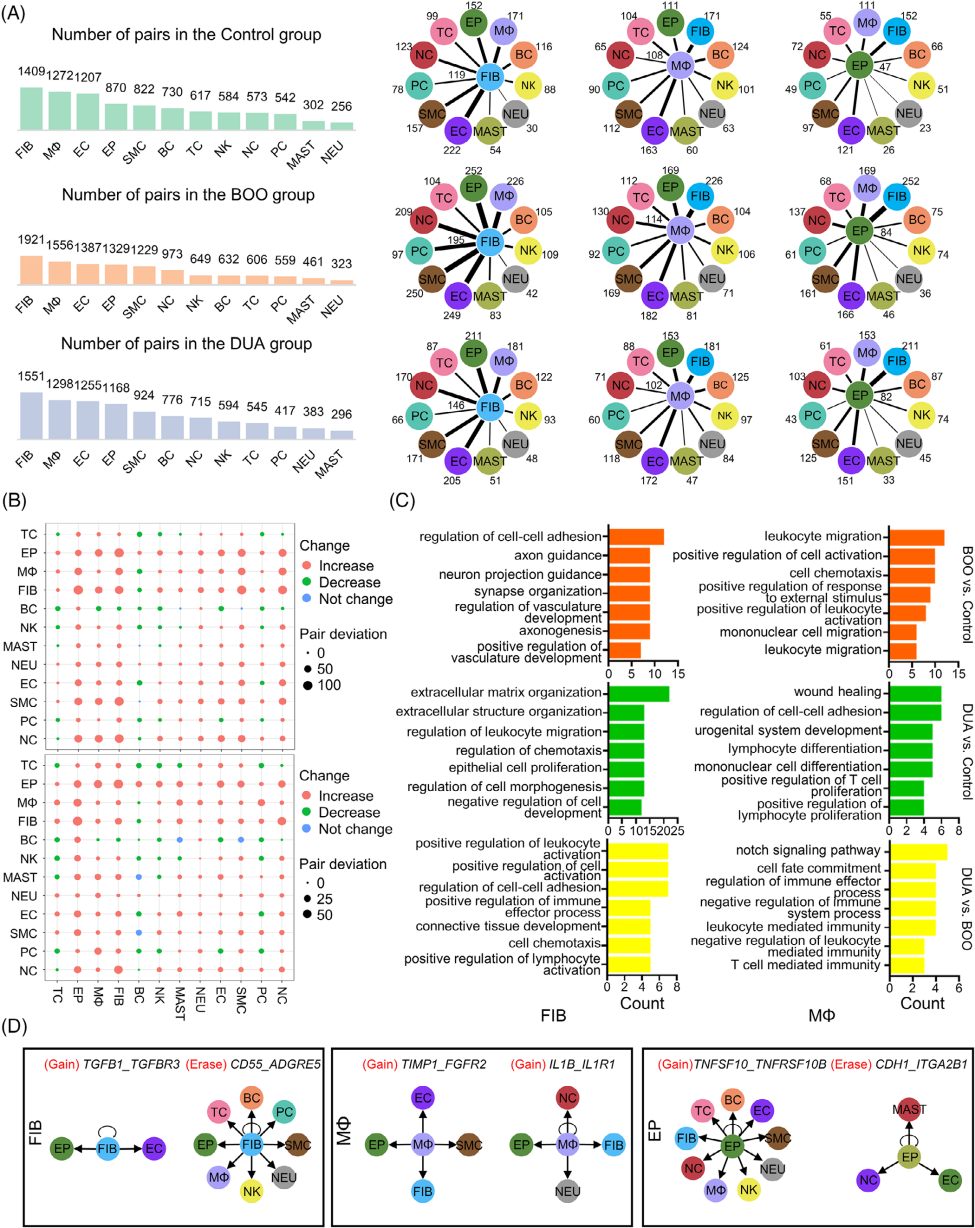
Cell–cell communication analysis based on Cellphone DB and further enrichment analysis. (A) Histogram shows the number of ligand–receptor pairs across different cell types in the three groups (left). Detailed view of the number of pairs from one cell type to another types (right, only displaying the top four cell types). The value next to the cell name represents the number of receptor ligand pairs. BOO, bladder outlet obstruction; DUA, detrusor underactivity; TC, T cell; EP, epithelial cell; MΦ, macrophage; FIB, fibroblast; BC, B cell; NK, nature kill cell; NEU, neutrophil; MAST, mast cell; EC, endothelial cell; SMC, smooth muscle cell; PC, plasma cell; NC, neural cell. (B) Dot plot displays relative changes in ligand–receptor pairs in each subtype in the BOO (top) and DUA (bottom) compared with control groups. Colors represent the change of them: red, increase; green, decrease; blue, no significant change. The size of dot represents the count of pair deviation. (C) Enrichment analysis (biological process) of altered ligand–receptor pairs. Colors show different comparisons, orange, BOO versus control; green, DUA versus control; and yellow, DUA versus BOO. (D) Network visualization of representative ligand–receptor pairs among different cell types between control and dysfunctional bladder.

Besides changes in gene expression and function within individual cell types, intracellular interactions also play a pivotal role in various biological processes.^19^ To delineate the intracellular communication network in bladder dysfunction, Cellphone DB analysis was employed to evaluate the number of ligand–receptor pairs. Notably, fibroblasts, macrophages, endothelial cells, and epithelial cells participated in the prominent intracellular communications with each group (Figure 3A). Compared with the control group, the numbers of ligand–receptor pairs in both BOO and DUA group were increased, indicating more complex cellular interactions in dysfunctional bladder (Figure 3A). However, at the group level, the top 20 ligand‐receptor pairs of each group were similar and macrophages dominated interactions with other cells depending on the CD74 molecule (Figure S3A). The further enrichment analysis of ligands and receptors in each group revealed no significant changes in the functional profile among the three groups. The roles of ligands and receptors in three groups were enriched in terms such as chemotaxis, calcium homeostasis, and the MAPK pathway, which are involved in fundamental physiological processes including cell migration, signaling, and proliferation (Figure S3B).

Hence, to mitigate the influence of those ligand–receptor pairs above, compared with the control group, we further investigated altered ligand‐receptor pairs in the BOO and DUA. In both groups, the interactions of fibroblasts, macrophages, and epithelial cells with most other cell types were significantly increased (Figure 3B). Subsequently, GO enrichment analysis using the altered ligand–receptor pairs was performed to reveal the function change of them. Interestingly, besides ECM remodeling, the increased ligand–receptor pairs of fibroblasts involved in the immune regulation and epithelial cell proliferation in the DUA group, which implied that fibroblasts might play a central role in the obstruction‐induced DUA (Figure 3C). As for macrophages, the function of increased ligand‐receptor pairs accumulated in the leukocyte migration and cell chemotaxis in the BOO group, whereas in the DUA group, those increased pairs involved in the process on tissue/organ healing and development. Such results indicated macrophages could regulate the inflammation and mediate bladder remodeling in the progress of obstruction‐induced bladder dysfunction (Figure 3C). Similar to the fibroblasts, the increased ligand–receptor pairs of epithelial cells were also associated with immune regulation and ECM organization in the DUA group (Figure S3C). As for representative ligand–receptor pairs, fibroblast gained TGFB‐TGFBR3, which are responsible for ECM remodeling. Similarly, both macrophage and epithelial cells gained ligand–receptor pairs associated with inflammation regulation, like IL1B‐IL1R1 and TNFSF10_TNFRSF10B (Figure 3D).

In summary, the cell–cell communication network of bladder dysfunction was established, highlighting the pivotal roles of fibroblasts, macrophages, and epithelial cells in regulating the progression of obstruction‐induced bladder dysfunction.

### 2.4 RBFOX1^+^ fibroblasts mediated ECM remodeling in the bladder dysfunction

**FIGURE 4:**
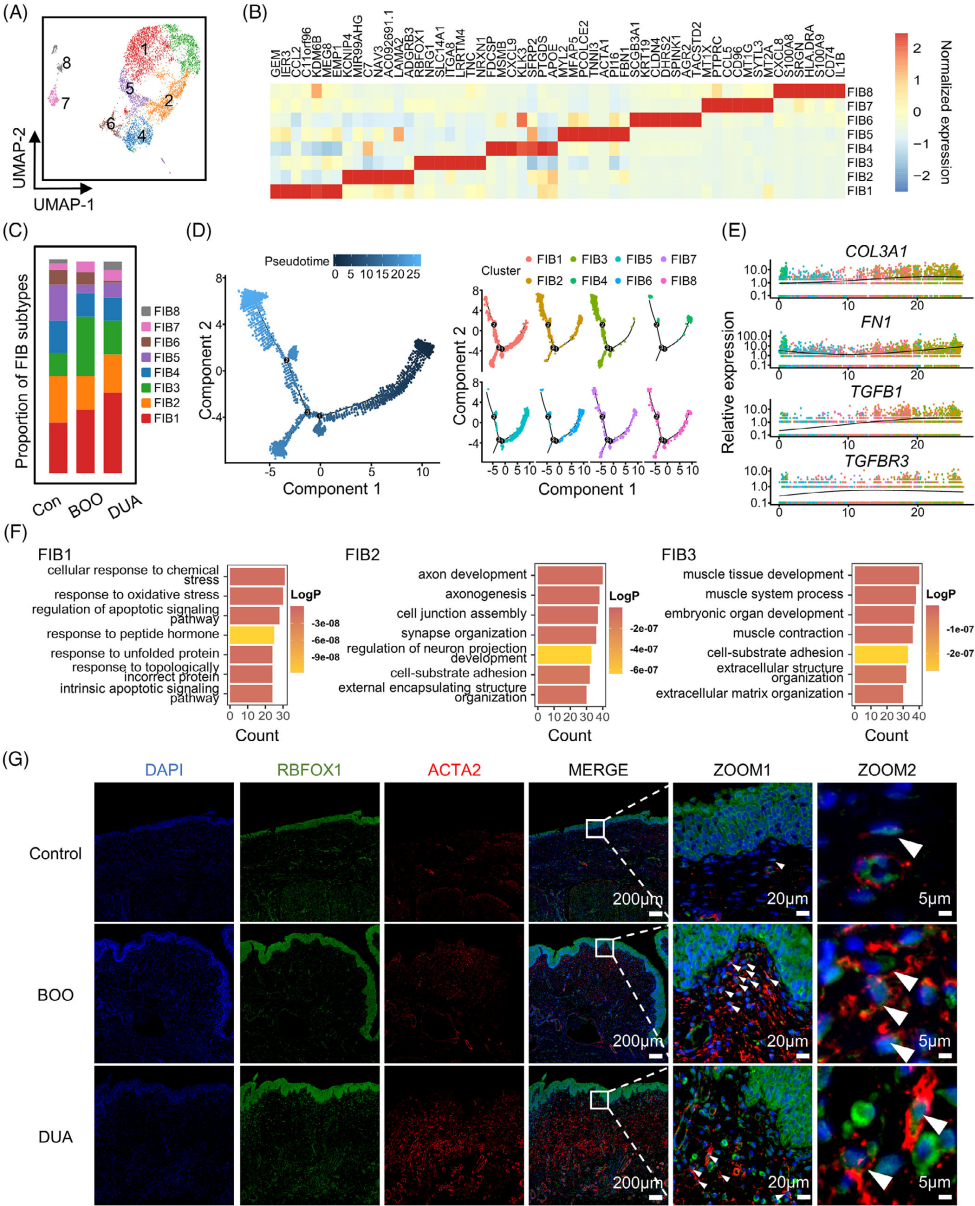
Subclustering and functional analysis revealed the key fibroblast subtype. (A) Uniform Manifold Approximation and Projection (UMAP) plot shows eight subtypes of fibroblasts identified after re‐cluster, colored according to cell types. (B) Heatmap shows the relative expression of top seven differentially expressed genes in each subtype. FIB, fibroblast. (C) Bar plot displays proportion of eight fibroblasts in the three groups. Con, control; BOO, bladder outlet obstruction; DUA, detrusor underactivity. (D) Pseudotime trajectory analysis of all fibroblasts, color‐coded by pseudotime (left), splitting by subtypes, colored according to cell subtypes (right). (E) The representative expression of genes related to organ remodeling among pseuotime. Different colored dots represent different single cell. (F) Enrichment analysis (biological process) of differentially expressed genes of fibroblasts 1, 2 and 3, colored according to the significance of each term. (G) Immunofluorescence staining was performed to localize RBFOX^+^ fibroblast in each group. Scar bar = 200 μm. The contents of the white box in MERGE are zoomed in the right as ZOOM1. White arrows point RBFOX and ACTA2 double‐positive cells. Scar bar = 20 μm. ZOOM2 shows representative double‐positive cells in the ZOOM1. Scar bar = 5 μm.

To further explore the heterogeneity and function of fibroblasts in bladder remodeling, we further re‐clustered them and identified eight distinct subclusters based on their expression of DEGs (Figure 4A,B). Fibroblasts 1–3 collectively constitute over half of the total fibroblasts in each group (Figure 4C). Of note, only fibroblasts 1 and 3 accounted for a higher ratio in the dysfunctional group than the control group (Figure 4C). GEM^+^ fibroblast 1 was incrementally increased from control to DUA, while RBFOX1^+^ fibroblast 3 had a higher ratio in the BOO group. KCNIP4^+^ fibroblast 2 and MYL2^+^ fibroblast 5 was significantly decreased in both BOO and DUA group (Figure 4C). Furthermore, pseudotime trajectory analysis was performed to illustrate the developmental trajectory of such eight subclusters (Figure 4D). According to it, fibroblasts 1, 2, 7, and 8 were uniformly distributed in each stage of the development trajectory, fibroblast 3 was mainly distributed in the terminal stage, and fibroblasts 4, 5, and 6 were mostly distributed in the initial stage (Figure 4D). The expression of genes associated with ECM organization gradually increased along the pseudotime trajectory and enriched in fibroblasts 2 and 3 subtypes (Figure 4E). Moreover, the gene expression profiles also indicated that these two subtypes predominately expressed ECM‐related genes (Figure S4A). However, subsequent GO enrichment analysis of DEGs of each fibroblast subtype showed that fibroblast 3, rather than fibroblast 1 or 2, participated in the regulation of ECM remodeling (Figure 4F). Considering alterations in the ratio, gene expression, and developmental status, RBFOX1^+^ fibroblast 3, a subtype that was increased in dysfunctional bladder, exhibited a distinct gene profile expression associated with ECM organization, and was located in the terminal developing state, could be a crucial subtype of fibroblasts mediating bladder remodeling during the progress of bladder dysfunction. The further immunofluorescence of bladder tissue was conducted and validated the presence of RBFOX1^+^ fibroblast 3 (Figure 4G). RBFOX1 and ACTA2 (marker for fibroblast) double‐positive cells were mainly localized in the lamina propria and increased in both BOO and DUA group (Figure 4G), which was consistent with the aforementioned changes in cellular ratios.

### 2.5 Tissue inhibitors of metalloproteinase (TIMP)‐related macrophages increased in the bladder dysfunction

**FIGURE 5:**
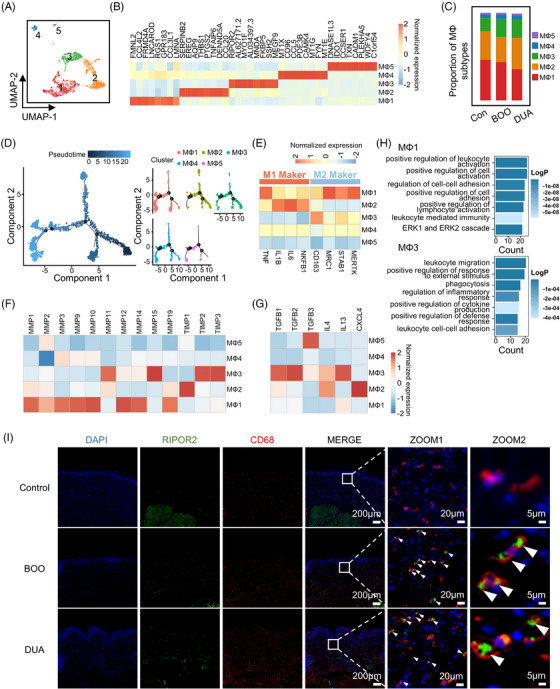
Subclustering and functional analysis revealed the key macorphage subtype. (A) Uniform Manifold Approximation and Projection (UMAP) plot shows five subtypes of macrophages identified after re‐cluster, colored according to cell types. (B) Heatmap shows the relative expression of top seven differentially expressed genes in each subtype. MΦ, macrophage. (C) Bar plot displays proportion of five macrophages in the three groups. Con, control; BOO, bladder outlet obstruction; DUA, detrusor underactivity. (D) Pseudotime trajectory analysis of all macrophages, colored according to the pseudotime (left), splitting by subtypes, colored according to cell subtypes (right). (E–G) Heatmap shows relative expression of M1 macrophage and M2 macrophage marker genes (E), MMP/TIMP gene family (F), and type 2 cytokine genes (G) in macrophage subtypes. (H) Gene ontology (GO) enrichment analysis (biological process) of differentially expressed genes of macrophages 1 and 3, colored according to the significance of each term. (I) Immunofluorescence staining was performed to localize RIPOR2^+^ macrophage in each group. Scar bar = 200 μm. The contents of the white box in MERGE are zoomed in the right as ZOOM. White arrows point RIPOR2 and CD68 double‐positive cells. Scar bar = 20 μm. ZOOM2 shows representative double‐positive cells in the ZOOM1. Scar bar = 5 μm.

Five distinct subtypes of macrophages were identified after re‐cluster (Figure 5A,B). Among them, macrophages 1, 2, and 3 constituted more than 90% of the total cell population (Figure 5C). Macrophage 1 was gradually decreased from control to DUA. Macrophage 2 did not show significant trend of change, while macrophage 3 was increased in both BOO and DUA group. Further pseudotime trajectory analysis showed that macrophage had more complex development trajectory (Figure 5D). Macrophages 1 and 5 were mainly distributed in the terminal stage of development trajectory, while other subtypes had no obvious distribution characteristics (Figure 5D). Considering the distinct functions of various subtypes of macrophages in tissue injury and fibrotic remodeling,^20^ we attempted to categorize the five subtypes of macrophages based on the defined marker genes of M1 (TNF, IL1B, and NFKB1) and M2 (CD163, MRC1, STAB1, and MERTK).^21^, ^22^ In this manner, macrophage 2 was M1‐like and macrophage 3 was M2‐like. However, macrophage 1 highly expressed both M1 and M2 maker genes, whereas macrophages 4 and 5 rarely expressed both marker genes (Figure 5E). Hence, further expression analysis was conducted on several genes previously implicated in immune regulation and organ remodeling, such as matrix metalloproteinase (MMP) and TIMP family genes,^23^, ^24^ TGFB family genes, as well as type 2 cytokines, to evaluate the functions of each subtype on bladder remodeling (Figure 5F,G). Intriguingly, RIPOR2^+^ macrophage 3 showed a high expression of genes associated with TIMPs, TGFB, and type 2 cytokines. Furthermore, the functional analysis further confirmed that subtype could play a crucial role in mediating bladder dysfunction through pathways related to above‐mentioned genes, whose functions were primarily enriched in the terms of immune regulation (Figure 5H and Figure S5A). The immunofluorescence confirmed the existence of RIPOR2^+^ macrophage in both BOO and DUA groups (Figure 5I). Furthermore, in accordance with a previous study,^13^ we observed an increased infiltration of macrophages (identified by CD68 staining) into both the epithelium and lamina propria (Figure 5I).

### 2.6 UPK^+^ umbrella cells (UCs) decreased and SHH^+^ basal cells (BCs) increased in bladder dysfunction

**FIGURE 6:**
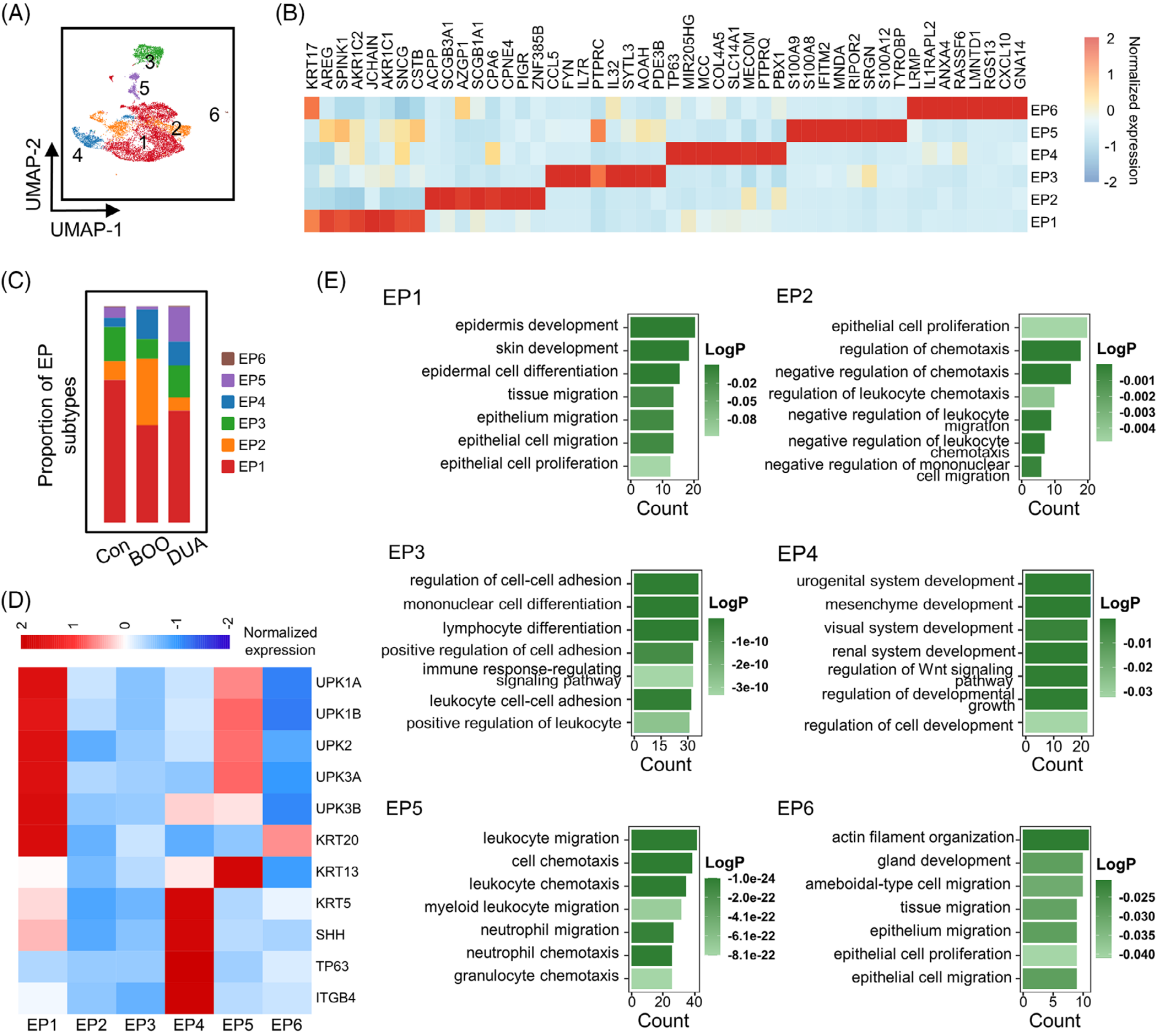
The change of subtypes and functions of epithelial cells during bladder dysfunction. (A) Uniform Manifold Approximation and Projection (UMAP) plot shows six subtypes of epithelial cells identified after re‐cluster, colored according to cell types. (B) Heatmap shows the relative expression of top eight differentially expressed genes in each subtype. EP, epithelial cell. (C) Bar plot displays proportion of eight epithelial cells in the three groups. Con, control; BOO, bladder outlet obstruction; DUA, detrusor underactivity. (D) Heatmap shows the relative expression of marker genes for anatomical classification of epithelial cells in each subtype. (E) Gene ontology (GO) enrichment analysis (biological process) of differentially expressed genes of all subtypes of epithelial cells, colored according to the significance of each term.

As the barrier and sensor of the bladder, urothelium plays a pivotal role in maintaining the normal function of the bladder. Further subcluster of epithelial cells identified six subtypes (Figure 6A,B). Since the urothelium is anatomically stratified into three distinct layers^26^: the UCs, marked by uroplakin (UPK) gene family (UPK1A, UPK1B, UPK2, UPK3A, and UPK3B) and KRT20,^27^, ^28^ intermediate cells (ICs) marked by KRT13 and low‐expressed UPK gene family,^27^ and BCs, marked by KRT5, SHH, TP63, and ITGB4^27^, ^28^, ^29^, ^30^; we used the markers of three subtypes to further identify these six subtypes (Figure 6D). The predominant subtype among six epithelial cells, epithelial cell 1, was identified as UC, whose functions were associated with epithelium migration (Figure 6C,D). Relevant to its developmental functions, epithelial cell 4 demonstrated a high expression profile of marker genes associated with BC (Figure 6D,E). The epithelial cell 5 was identified as IC and with the function of the immune regulation (Figure 6D,E). The epithelial cells 2 and 3, although they could not be classified into the aforementioned three subtypes, exhibited a similar functional profile to that of epithelial cell 5 (Figure 6E). Taken together, the UPK and KRT20 positive UCs (epithelial cell 1) were decreased, while SHH^+^ BCs (epithelial cell 4) were increased in the bladder dysfunction, indicating damage of the bladder barrier and concomitant compensatory proliferation of BCs. Meanwhile, other cell types increased in BOO or DUA group, such as epithelial cells 2 and 5 were involved into immune regulation.

## 3 DISCUSSION

DUA, with a high prevalence^3^, ^4^ and deleterious impact on patients’ health,^2^ has always been an urgent problem to be solved in urology. Unfortunately, the lack of effective interventions in clinical practice persists due to the progressive nature of bladder injury and fibrosis, which often results in irreversible damage to bladder function. The challenges of such condition arise from a limited comprehension of its underlying pathophysiological mechanism. Hence, it is highly desirable to investigate the molecular mechanisms underlying DUA to provide more support for further research and clinical decision‐making. In this study, we collected bladder samples from control, BOO, and BOO‐induced DUA patients and constructed the first single‐cell transcriptomic atlas of obstruction‐induced bladder remodeling. Among normal and remodeled bladder, 12 cell types were identified, which showed significant heterogeneity in both cellular composition and functional characteristics. Importantly, we identified the DEGs in each group and observed an enrichment of immune‐related processes in the stage of BOO, while fibrosis‐related functions were found to be prevalent in DUA. Interestingly, we have identified a noteworthy genes, SCGB1A1 (the second DEG in the BOO group), which were reported as potential biomarkers for mechanical ventilation‐induced lung injury.^31^ Given the presence of a similar mechanical environment characterized by pressure overload in the bladder following obstruction, this gene has the potential to serve as a biomarker for obstruction‐induced bladder injury, which warranted further investigation. The subsequent analysis of DEGs in specific cell types showed that biological processes on immune and inflammation regulation were enriched in macrophages and epithelial cells, while fibroblasts demonstrated function associated with ECM remodeling.

Cell–cell communication analysis unveiled that cellular interactions were significantly altered, indicating the remodeling of intercellular regulation during bladder dysfunction. Fibroblasts, macrophages, and epithelial cells emerged as the primary cell types exhibiting significant intercellular interactions across all three groups, and their intercellular communications were markedly increased in both BOO and DUA groups. Additionally, interactions regarding ECM organization, such as TGFB‐TGFBR3,^18^ were gained in the fibroblasts. Macrophages and epithelial cells exhibited interactions pertaining to inflammation regulation, like IL1B‐IL1R1^32^ and TNFSF10_TNFRSF10B.^33^ These findings were in line with the functional profiles of cell‐specific DEGs in that three cell types. Collectively, fibroblasts, macrophages, and epithelial cells could promote inflammation and fibrosis through their altered cellular interactions.

Consider the potentially critical role of three types of cells in bladder dysfunction, further re‐clustering and subsequent analysis of them were performed. The RBFOX1^+^ fibroblast, primarily involving in ECM remodeling, was located in the lamina propria and exhibited increased abundance in dysfunctional bladder. Their location and alteration of ration and function indicated that they could be the key fibroblast mediating bladder remodeling. Intriguingly, we found RIPOR2^+^ macrophage could be a crucial macrophage subtype involved in the regulation of immune and inflammation through TIMPs, type 2 cytokines, and TGF‐β,^34^ instead of classical M1/2 polarization, in the obstruction‐induced DUA. As for epithelial cells, notably, we found a novel role of epithelial cells in the regulation inflammation and immune responses in bladder dysfunction, which was disregarded in previous studies. Meanwhile, the dynamic changes of UPK^+^ UCs, SHH^+^ BCs, and inflammatory epithelial cells indicated the coexistence of superficial epithelial injury, basal repair, and epithelial inflammation in the obstruction‐induced DUA.

Taken together, this study constructed a comprehensive single‐cell transcriptomic atlas to elucidate cellular heterogeneity and functional alterations in obstruction‐induced bladder remodeling. Furthermore, the altered DEGs and intercellular interactions revealed the obstruction‐induced bladder dysfunction is a process from injury, inflammation to fibrosis.^6^ Hence, early removal of obstruction to alleviate bladder injury and subsequent control of inflammation and immune response could be a feasible approach to alleviate bladder fibrosis and dysfunction. Specifically, the RBFOX1^+^ fibroblast, RIPOR2^+^ macrophage mediating the TIMPs, type 2 cytokines and TGF‐β‐related pathways, and urothelial damage and inflammation could be potential targets warranted further investigations. Hence, these findings could offer an in‐depth insight for pathophysiological process of obstruction‐induced DUA and promising targets for prevention or treatment of this disorder.

## 4 MATERIALS AND METHODS

### 4.1 Study population

The determination of obstruction‐induced bladder dysfunction was based on the comprehensive assessment of symptoms, scales (International Prostate Symptom Score and QoL), imaging, and urodynamics in accordance with the European Association of Urology guidelines.^35^ Specifically, all patients with LUTS (including frequency, urgency, dysuria, etc.), ultrasound/MRI‐measured enlarged prostate, and bladder dysfunction based on urodynamics were eligible for this study. The participants were further categorized into BOO and DUA groups based on the urodynamic test results. The patients in the BOO group exhibited a urodynamic pattern characterized by an increased detrusor pressure and reduced urinary flow during voiding, along with a bladder outlet obstruction index exceeding 40 cm H_2_O (BOOI = PdetQmax – 2Qmax).^36^ Similarly, the patients with a low pressure and flow urodynamic pattern and a bladder contractility index (BCI = PdetQmax + 5Qmax) of less than 100 were categorized as DUA group.^37^ Male patients diagnosed with bladder tumor but exhibiting normal bladder function were chosen as the control group. Exclusion criteria included a history of prior radiotherapy, chemotherapy, intravesical operations, urinary infection, or any other conditions that may lead to structural or functional impairment of the bladder (Figure S1D). All eligible participants had provided informed consents. This study was performed with the approval of the Medical Ethical Review Committee of West China Hospital of Sichuan University (20201282).

### 4.2 Sample collecting

Partial‐thickness (mucosa to superficial muscularis) bladder tissues with the diameter of about 1 cm were obtained during the operation. For patients with bladder dysfunction (including BOO and DUA), extra bladder tissue was collected during transurethral resection of prostate. To avoid the effect of tumor, normal bladder tissue was obtained at least 2 cm away from the tumor tissue as controls during transurethral resection of bladder tumor. Furthermore, the postoperative histopathological examination of the specimens yielded supplementary evidence confirming their normalcy. The isolated tissues were promptly transferred to the laboratory in ice‐cold DMEM (Gibico, Cat: 11995040) to the laboratory for subsequent processing. The preparation of bladder single‐cell suspension for scRNA‐seq was described in a previous study.^11^ Specifically, after washing with 4°C DPBS for two times, bladder tissues were cut into pieces using surgical scissors and digested with 10 mL of collagenase type I (1.5 mg/mL, Roche, Cat: 10103578001) and DNase I (0.2 mg/mL; Sigma‐Aldrich, Cat: DN25) for 30 min at 37°C water bath. Subsequently, the digestion was terminated by adding an equal volume of DMEM supplemented with 10% FBS (Gibico, Cat: 10099141). After centrifugation at 300 × *g* for 5 min at 4°C, supernatant was discarded. Then, the red blood cells were removed by adding 2 mL of pre‐cooled red blood cell lysis buffer (Solarbio, Cat: R1010), followed by centrifugation at 300 × *g* for 5 min at 4°C. Next, sediments were resuspended with 4°C DPBS and filtered through a 40‐μm cell strainer to obtain single‐cell suspension. Finally, the count and viability of cells were assessed using a hemocytometer with trypan blue staining (Solarbio, Cat: C0040). The subsequent single‐cell sequencing necessitated the use of single‐cell suspensions with a live cell ratio exceeding 80%.

### 4.3 10× Single‐cell library construction and sequencing

Single‐cell suspensions were converted to barcoded libraries based on the standard protocols of the 10 × Genomics Chromium Single Cell 3′library and Gel Bead Kit (version 3.1) to capture 10,000 cells per chip. All procedures including the library construction followed the 10 × Genomics protocols.^14^ The libraries were sequenced on the Illumina HiSeq X Ten platform (CapitalBio Technology) using 150 base‐pair paired‐end reads.

### 4.4 Quality control and cell clustering

The raw sequencing data were processed by Cell Ranger (version 5.0.0) to generate gene expression matrices. Such matrices were read into the Seurat R package for the further processing.^38^ Qualitied cells with unique molecular identifier (UMI) numbers > 1000, 200 < gene numbers < 5000, and <10% mitochondrial‐derived UMI counts were retained for further analysis. To address the batch effect, harmony was applied for the batch integration.^39^, ^40^ The dimensionality of the scRNA‐Seq data was reduced by principal component analysis (PCA). “FindClusters” function with an optimal resolution was used to identify the main cell clusters. To visualize the data, UMAP was employed. Finally, canonical markers, including T cells (CD3D and CD3E), epithelial cells (KRT18 and KRT19), macrophages (CD68), fibroblasts (COL3A1 and LUM), B cells (CD79A and MS4A1), natural killer cells (NKG7 and GNLY), neutrophils (FCGR3B), mast cells (CPA3 and TPSAB1), endothelial cells (VWF), smooth muscle cells (MYH11), plasma cells (MZB1), and nerve cells (NRXN1), were used to annotated known biological cell types. Subsequently, certain cell types were clustered into subclusters for further analysis.

### 4.5 DEGs analysis

DEGs among the three groups were identified by R package edgeR with a significance threshold of adjusted *p* value (Benjamini–Hochberg method) <0.05 and |log_2_FC| > 0.5. Subsequently, GO analysis was conducted to explore the functional annotations of these significant DEGs.

### 4.6 Cell–cell communication analysis

Cell–cell communication networks among different cell types were explored using Cellphone DB, a database of ligands, receptors, and their interactions.^19^ The significant ligand–receptor pairs were defined as those with a *p* value < 0.05. The cell–cell interaction networks were visualizing the significant ligand–receptor pairs between each pair of cell types. The function of these significant ligands and receptors was further investigated using GO analysis.

### 4.7 Pseudotime trajectory analysis

Pseudotime trajectory analysis by Monocle2^41^ were performed to depict the developmental trajectory of cell subtypes. Meanwhile, “differentialGeneTest” function were applied to find significantly changed genes with *q* value < 0.01.

### 4.8 Histologic staining and immunofluorescence

The bladder samples were harvested and fixed in 4% paraformaldehyde for 24 h. Subsequently, they were embedded in paraffin and sectioned into 5 μm slices. The slices were subjected to H&E staining to visualize the histological and morphological or Masson's trichrome staining to demonstrate collagen deposition. For immunofluorescence, the sections were stained using antibodies against RBFOX1 (1:100, 22647‐1‐AP; Proteintech), ACTA2 (1:250, ab7817; Abcam), RIPOR2 (1:100, PA5‐97196; Invitrogen), and CD68 (1:200, 97778; Cell Signaling Technology). All sections were imaged by a digital camera (3DHISTECH, Case Viewer 2.4).

### 4.9 Statistical analysis

All statistical analyses were performed by R (version 4.0.1). A two‐tailed *p* value < 0.05 was considered statistically significant.

## AUTHOR CONTRIBUTIONS

Jiawei Chen, Deyi Luo, and Banghua Liao designed the study. Jiawei Chen and Liao Peng recruited patients. Jiawei Chen, Liao Peng, and Guo Chen performed the experiments, analyzed the data, and wrote the article. Yuanzhuo Chen and Chi Zhang collected the bladder sample. Xiao Zeng and Jie Zhang performed the urodynamic testing. Hong Shen, Banghua Liao, and Deyi Luo supervised study and revised manuscript. All authors have read and approved the final manuscript.

## CONFLICT OF INTEREST STATEMENT

The authors declare no conflicts of interest.

## ETHICS STATEMENT

This study was approved by the Medical Ethical Review Committee of West China Hospital of Sichuan University (20201282) and had a clinical registration (Registration number: ChiCTR2100042928). This study was performed in accordance with the Declaration of Helsinki. All participants provided informed consents.

## Supporting information

Supporting information

## Data Availability

The data in this study are available from the corresponding author upon reasonable request.
