# Supplementary material for: Single‐cell transcriptomics reveal the remodeling landscape of bladder in patients with obstruction‐induced detrusor underactivity
Source: MedComm (2020). 2024 Feb 26;5(3):e490. doi: 10.1002/mco2.490 (PMC10896249; doi:10.1002/mco2.490)
Supplement: Supplementary file 1 — Supporting information [file MCO2-5-e490-s001.pdf]

**Title:** Single-cell transcriptomics reveal the remodeling landscape of bladder in patients with obstruction-induced detrusor underactivity

**Running title:** Single-cell atlas of bladder remodeling

**Author:** #Jiawei Chen[1,2]; #Liao Peng[1,2]; #Guo Chen[2,3]; Yuanzhuo Chen[1,2]; Xiao Zeng [1,2]; Jie Zhang[1,2]; Chi Zhang[1,2]; Hong Shen[1,2]; *Banghua Liao[1,2]; *Deyi Luo[1,2,4]
#Jiawei Chen, Liao Peng and Guo Chen contributed equally to this study should be considered as co-first author.

**Authors affiliation:**

1. Department of Urology, West China Hospital, Sichuan University, Chengdu, Sichuan, China
2. Department of Urology, Institute of Urology, West China Hospital, Sichuan University, Chengdu, Sichuan, China
3. Department of Urology and Pelvic surgery, West China School of Public Health and West China Fourth Hospital, Sichuan University, Chengdu, Sichuan, China
4. Pelvic Floor Diseases Center, West China Tianfu Hospital, Sichuan University, Chengdu, Sichuan, China.

**Corresponding author:**

Banghua Liao: E-mail: liaobanghua@wchscu.cn; Tel: +86-180-9006-1627

Deyi Luo: E-mail: luodeyi1985@163.com; Tel: +86-189-8060-6809 Fax: +86-28-8542 2451 ORCID: 0000-0002-9436-036X

**Figure S1 Gene expression, functional analysis and cellular heterogeneity of each cell types. A** Heatmap shows the relative expression of top 20 differentially expressed genes in each cell types. Colors on the top represent cell types (right). The chart shows the number and proportion of each subtype in the three groups (right). TC, T cell; EP, epithelial cell; MΦ, macrophage; FIB, fibroblast; BC, B cell; NK, nature kill cell; NEU, neutrophil; MAST, mast cell; EC, endothelial cell; SMC, smooth muscle cell; PC, plasma cell; NC, neural cell. **B** Bubble chart shows the results of GO enrichment analysis (biological process) according to DEGs of each cell types. The size of bubble represents the count of genes in that term, and colors display the significance. **C** UMAP plots of the 12 cell types in the three groups. Pie chart on the upper right shows the proportion of each cell types. Colors represents different cell types (on the right). **D** Flowchart of study populations. NMIBC, non-muscle-invasive bladder cancer; LUTS, lower urinary tract symptom; IPSS International Prostate Symptom Score; QoL, quality of life; MRI, magnetic resonance imaging; BOOI, bladder outlet obstruction index; BCI, bladder contractility index; BOO, bladder outlet obstruction; DUA, Detrusor underactivity.

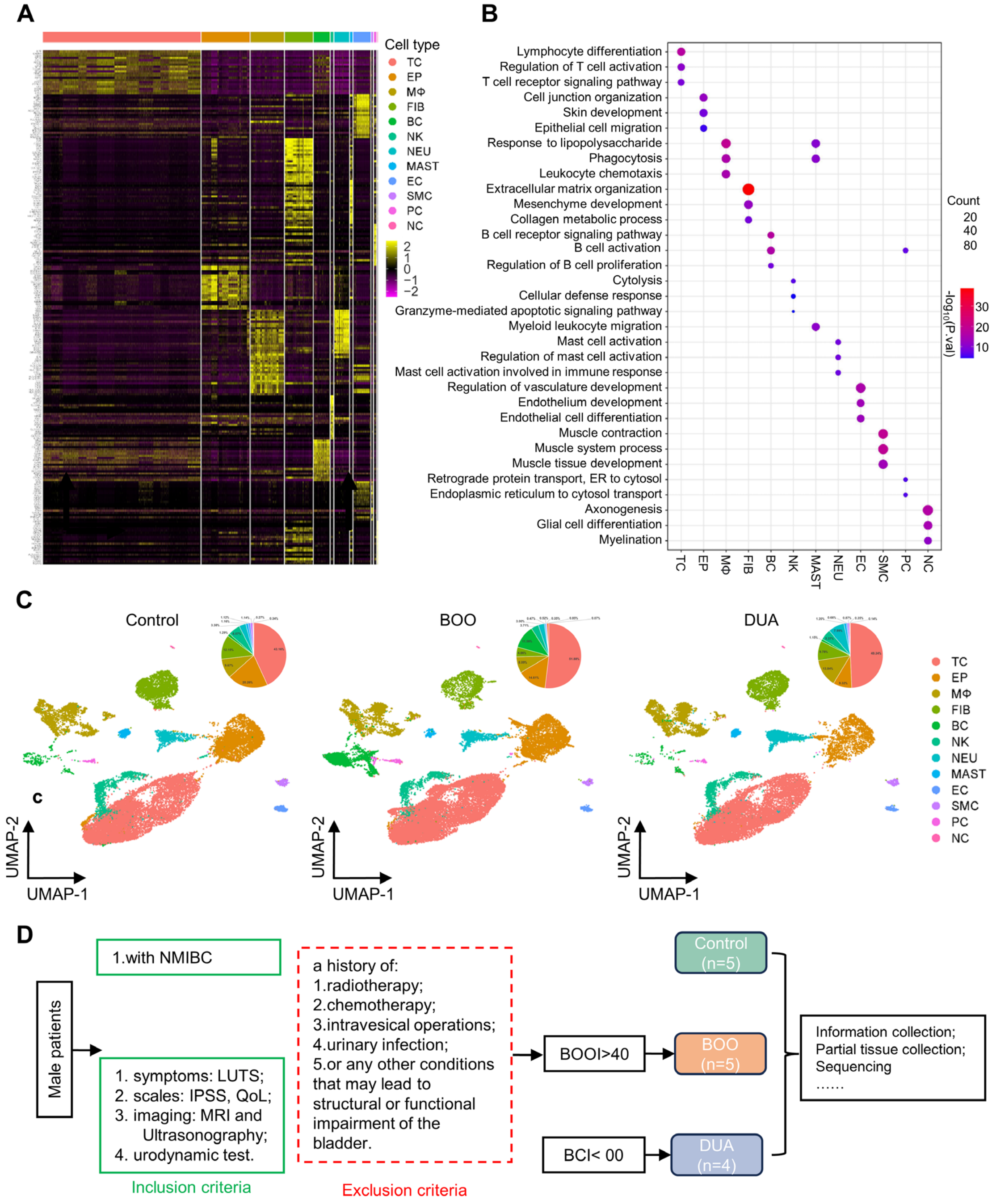
A
Cell type
TC
EP
MΦ
FIB
BC
NK
NEU
MAST
EC
SMC
PC
NC
2
1
0
−1
−2
B
Lymphocyte differentiation
Regulation of T cell activation
T cell receptor signaling pathway
Cell junction organization
Skin development
Epithelial cell migration
Response to lipopolysaccharide
Phagocytosis
Leukocyte chemotaxis
Extracellular matrix organization
Mesenchyme development
Collagen metabolic process
B cell receptor signaling pathway
B cell activation
Regulation of B cell proliferation
Cytolysis
Cellular defense response
Granzyme-mediated apoptotic signaling pathway
Myeloid leukocyte migration
Mast cell activation
Regulation of mast cell activation
Mast cell activation involved in immune response
Regulation of vasculature development
Endothelium development
Endothelial cell differentiation
Muscle contraction
Muscle system process
Muscle tissue development
Retrograde protein transport, ER to cytosol
Endoplasmic reticulum to cytosol transport
Axonogenesis
Glial cell differentiation
Myelination
TC EP MΦ FIB BC NK MAST NEU EC SMC PC NC
Count
20
40
80
-log10(P.val)
30
20
10
C
Control
BOO
DUA
UMAP-2
UMAP-1
TC
EP
MΦ
FIB
BC
NK
NEU
MAST
EC
SMC
PC
NC
D
Male patients
1.with NMIBC
1. symptoms: LUTS;
2. scales: IPSS, QoL;
3. imaging: MRI and Ultrasonography;
4. urodynamic test.
Inclusion criteria
a history of:
1.radiotherapy;
2.chemotherapy;
3.intravesical operations;
4.urinary infection;
5.or any other conditions that may lead to structural or functional impairment of the bladder.
Exclusion criteria
BOOI>40
BCI< 00
Control (n=5)
BOO (n=5)
DUA (n=4)
Information collection;
Partial tissue collection;
Sequencing
……

**Figure S2 Gene function profiles in cell-specific manner in the dysfunction bladder.**

**A-B** Cell-specific GO enrichment analysis (biological process) according to the down-regulated differentially expressed genes of BOO (A) or DUA (B) compared with control, color-coded by the significance of each term. Colors on the top represent cell types (right). BOO, bladder outlet obstruction; DUA, detrusor underactivity. TC, T cell; EP, epithelial cell; MΦ, macrophage; FIB, fibroblast; BC, B cell; NK, nature kill cell; NEU, neutrophil; MAST, mast cell; EC, endothelial cell; SMC, smooth muscle cell; PC, plasma cell; NC, neural cell.

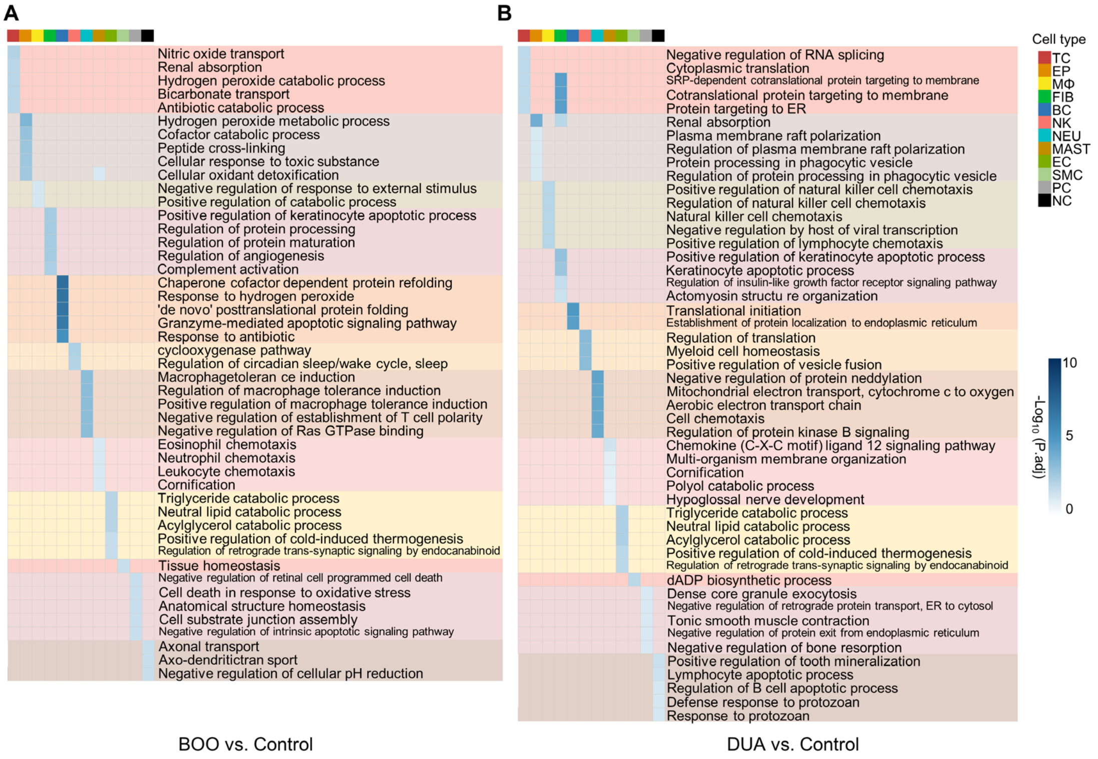

**Figure S3 The cell interactions between each cell and further functional analysis.** **A** Top 20 ligand-receptor pairs among three groups. The outer ring's color indicates the cell type, and the inner ring's color represents the ligand or receptor, green, ligands; blue, receptors. BOO, bladder outlet obstruction; DUA, detrusor underactivity, TC, T cell; EP, epithelial cell; MΦ, macrophage; FIB, fibroblast; BC, B cell; NK, nature kill cell; NEU, neutrophil; MAST, mast cell; EC, endothelial cell; SMC, smooth muscle cell; PC, plasma cell; NC, neural cell. **B** Bubble chart shows the results of GO enrichment analysis (biological process) according to ligands and receptors of three groups. The size of bubble represents the count of genes in that term, and colors display the significance. **C** GO enrichment analysis (biological process) of altered ligand-receptor pairs of epithelial cells. Colors show different comparisons, orange, BOO vs. control; green, DUA vs. control; and yellow, DUA vs. BOO.

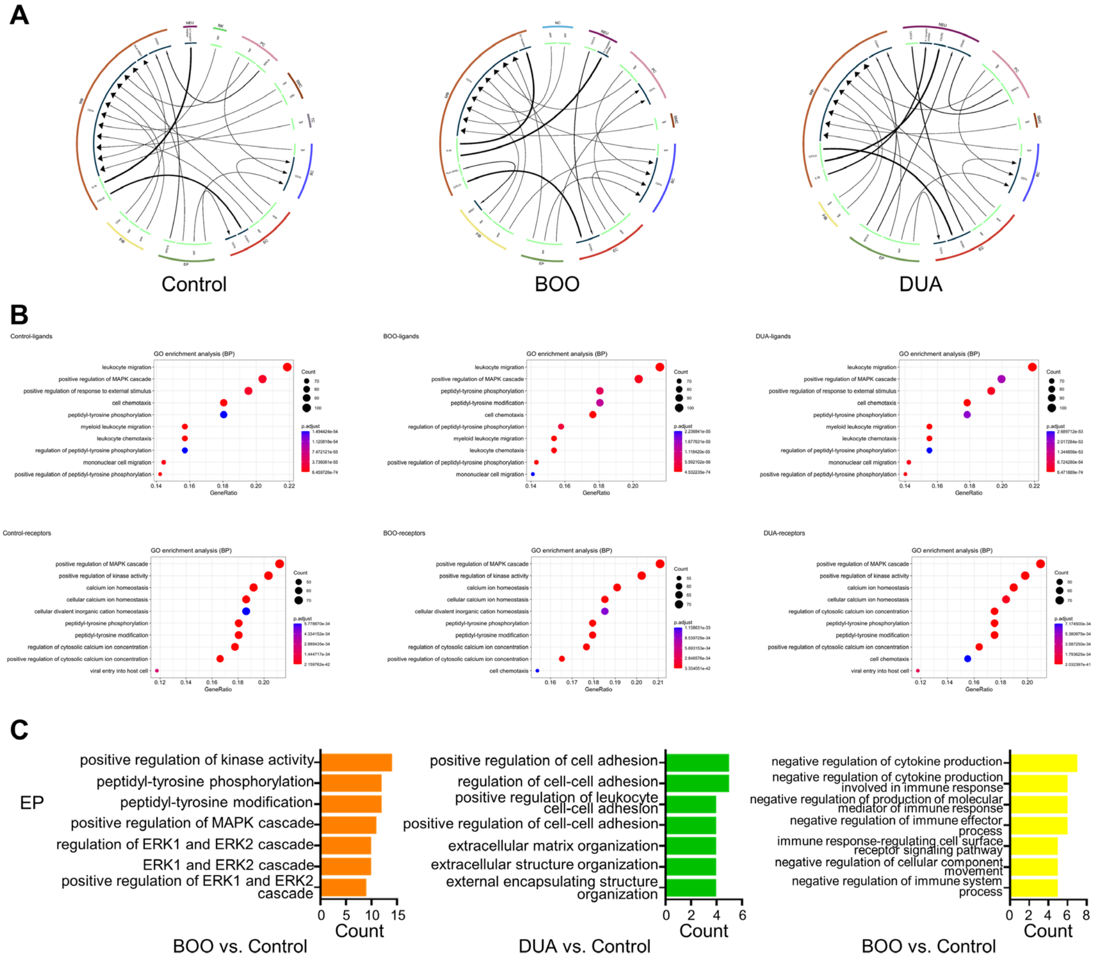
A
Control
BOO
DUA
B
Control-ligands
BOO-ligands
DUA-ligands
Control-receptors
BOO-receptors
DUA-receptors
GO enrichment analysis (BP)
GeneRatio
Count
p.adjust
C
EP
positive regulation of kinase activity
peptidyl-tyrosine phosphorylation
peptidyl-tyrosine modification
positive regulation of MAPK cascade
regulation of ERK1 and ERK2 cascade
ERK1 and ERK2 cascade
positive regulation of ERK1 and ERK2 cascade
Count
BOO vs. Control
positive regulation of cell adhesion
regulation of cell-cell adhesion
positive regulation of leukocyte cell-cell adhesion
positive regulation of cell-cell adhesion
extracellular matrix organization
extracellular structure organization
external encapsulating structure organization
Count
DUA vs. Control
negative regulation of cytokine production
negative regulation of cytokine production involved in immune response
negative regulation of production of molecular mediator of immune response
negative regulation of immune effector process
immune response-regulating cell surface receptor signaling pathway
negative regulation of cellular component movement
negative regulation of immune system process
Count
BOO vs. Control

**Figure S4 A** Heatmap shows the relative expression of representative extracellular matrix related genes in each subtype.

A

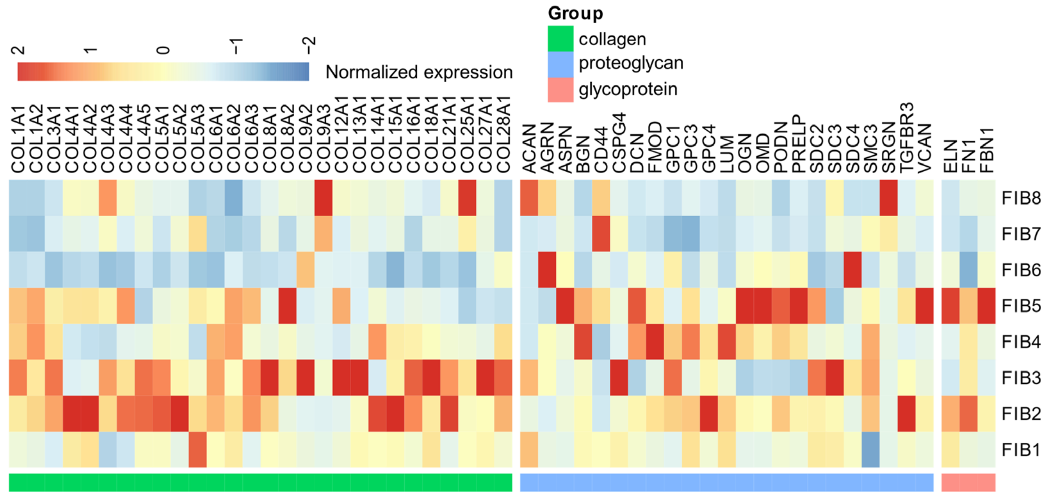

**Figure S5 A** Go enrichment analysis (biological process) of differentially expressed genes of macrophage-2, -4 and -5

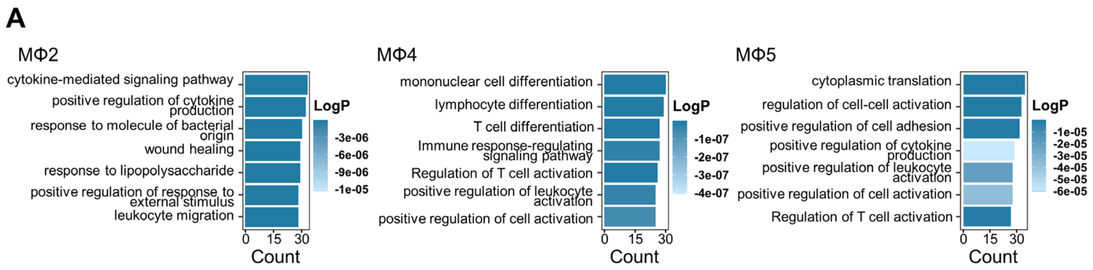

**Table S1** The urodynamic parameters of each patient

| Patient | BOOI | BCI |
|---|---|---|
| BOO-1 | 101 | 157 |
| BOO-2 | 77 | 147 |
| BOO-3 | 99 | 134 |
| BOO-4 | 78 | 113 |
| BOO-5 | 62 | 139 |
| DUA-1 | 13 | 20 |
| DUA-2 | 8 | 50 |
| DUA-3 | 5 | 61 |
| DUA-4 | 13 | 27 |

BOO = bladder outlet obstruction; DUA = detrusor underactivity; BOOI = bladder outlet obstruction index; BCI = bladder contractility index.
